# Teamwork in Airway Surgery

**DOI:** 10.3389/fped.2021.621449

**Published:** 2021-02-24

**Authors:** Martin J. Elliott, Derek Roebuck, Nagarajan Muthialu, Richard Hewitt, Colin Wallis, Paolo DeCoppi, Denise Macintyre, Clare Ann McLaren

**Affiliations:** ^1^University College London, London, United Kingdom; ^2^Perth Children's Hospital, Perth, WA, Australia; ^3^The Great Ormond Street Hospital for Children NHS Foundation Trust, London, United Kingdom

**Keywords:** teamwork, airway surgery, quality, pediatrics, trachea

## Introduction

The twentieth century saw the gradual disappearance of the heroic individual doctor and the emergence of specialities with distinct governance structures through colleges and societies. These defined training and issued qualifications. In our world, cardiothoracic surgery split from general surgery, pediatric surgery from general surgery and ear, nose and throat surgery emerged in parallel. The separation produced rapid advances in each field but, as an unexpected consequence, the disciplines grew apart, developing their own ways of working and their own tribal cultures. Our patients (and their conditions) did not recognize this, and they would find that the way in which their disease was treated varied widely–defined largely by the speciality with which they first came into contact.

The management of complex airway disease in children exemplifies these problems, but also offers a solution. In the late twentieth century, patients were referred to individual surgeons who applied the skills of their own discipline to varying, but imperfect effect. Inter-discipline referral was rare, and sometimes difficult because the geographic location of services had become separated to different hospital sites in previous years. Sadly, and as we all now know, affected children often had problems which crossed the constrained boundaries which we physicians had drawn up. Tracheal stenosis is often combined with cardiovascular anomalies and genetic abnormalities are frequent. Upper gastro-intestinal tract issues including swallowing problems abound. Patients attending one speciality were referred to another for consultation on a *transactional* basis. Indeed, in dominantly private healthcare systems, this remains the case, as it can increase incomes to all parties. This slows decision making, fails to integrate views effectively and weights decision making in favor of the physician to whom primary referral is made. Our primary aim as physicians is “first, do no harm.” As Hull and Sevdalis pithily stated (1) “Teams create safety,” and as we hope to outline in this paper, teamwork also improve outcomes, creates efficiency, reduces cost and promotes research. Achieving these goals is good for patients and for the wider healthcare system.

## Some Local History

In the United Kingdom in the 1980s and 90s, referrals for small children with long segment congenital airway stenosis (LSCTS) passed through a series of gateways to pediatric or cardiac intensive care units, largely because of the resuscitative skills held by the staff there. Surgery tended to default to cardiac surgical teams because of the high incidence of associated cardiovascular anomalies and the need for cardiopulmonary bypass for repair. The incidence is low, and so each center saw a tiny number of patients, and experience was hard to acquire. There were only a few short case series in the literature upon which to base treatment choices, and few contained sufficient detail to be confident about all the relevant technical details and none had any long-term data. Several techniques had been described for repair, but patch tracheoplasty dominated, and mortality rates were high.

At that time, relevant skills were distributed in such a way that individuals had to be consulted to manage specific problems. For example, endoscopic examination of the airway was largely done by ear, nose and throat (ENT) surgeons, cardiologists helped diagnose and manage cardiac issues, and the surgical reparative skills crossed boundaries. Intensive care was mandatory, but often seen as a “service” to other teams, and nursing was undervalued. Interventional radiology was embryonic, but increasingly seen to be relevant, and palliative care was only a consultative service. The interfaces between services were relatively formal; a *consultative* interface. As Reason pointed out many years ago (2), it is the interfaces which go wrong and lead to error because of failures in communication. Each discipline approached problems in its own way according to its own (often limited) experience.

Those of us involved in the care of these children decided that this was not good enough and ***everyone*** involved met in 2000 to work out how we might better deal with complex airway cases. It was the birth of the GOSH^1^ Tracheal Team, and a fantastic meeting of minds. Several key decisions were made;

- The team should comprise all those coming into contact with such patients on a regular basis. Namely, but in no specific order of importance, cardiothoracic surgeons, ENT surgeons, interventional radiologists, specialist and intensive care unit (ICU) nurses, intensivists, respiratory physicians, pediatric general surgeons, anesthetists, diagnostic radiologists, speech therapists, physiotherapists, administrative staff, data managers, radiographers, cardiologists, and interested researchers and junior staff in training.
- A leadership structure was created.
- ALL referrals with complex airway problems would be reviewed by the Tracheal Team at a weekly multi-disciplinary team meeting (MDT).
- ALL relevant decisions about both individual patient care and overall strategy would be taken at the MDT, recorded and stored in a database.
- LSCTS would be treated by slide tracheoplasty on cardiopulmonary bypass or extracorporeal membrane oxygenation (ECMO), and where possible, cardiac lesions would be repaired at the same time.
- The team should learn to cross-skill to avoid delays to patient care. Specifically, this related to skills in fibreoptic bronchoscopy and balloon dilatation.
- All outcomes would be published, and attempts would be made over time to centralize care in the UK if results justified it.
- Links would be created with other interested specialists throughout the world.

Within just a few years we observed a significant increase in referral, improved outcomes and a dramatic reduction in the cost of care (3). Such single center reporting is prone to bias and uncertainty as to the cause of the improvement, but we maintain that all of the above decisions contributed in some way. We also noted greater cohesion, smoother decision making, constructive discussion and general happiness in the mode of working. Several other teams emerged simultaneously, notably in Chicago and Cincinnati, from different origins, and also commented on the value of integrated teamwork [see discussion at the end of (3)].

The growth of referral accelerated our learning, and the rate of improvement of results changed with it. In 2005–6 the team applied for, and was granted, national status by the National Health Service (NHS), becoming the sole center recognized for the treatment of complex airway disease in children. Since then growth has been continuous, with referrals coming from all over the world, bringing with it new challenges and increasing complexity of cases. Research blossomed, ranging from diagnostic techniques through to quality of life assessment, cell biology and transplantation. Our team was successful, and its teamwork “worked.”

We are particularly proud of the way in which certain roles developed within the team. Specifically, and against some initial resistance by vested interests, the radiographer in our team was taught to undertake bronchoscopy and balloon dilatation, a skill which now makes her one of the world leaders in this field and a significant contributor to the literature. We will return to cross-skilling later.

## The Theory

Is our experience unique, or are the consequences of good teamwork replicable? There has been a great deal of work undertaken about the importance of teamwork in surgery, and an excellent review of the relevant background for surgeons by the Royal College of Surgeons of England (RCS). This can be found at www.rcseng.ac.uk/surgeons/surgical-standards/professionalism-surgery/gsp. It is widely accepted that good teamwork improves clinical performance (4), patient outcomes (4, 5), and the well-being and retention of staff (6, 7). The effect on performance appears to be present even in a limited part of the patient journey, i.e., in the operating room (8). The RCS report highlights the point that teams come together to perform specific tasks, and thus the membership of the team must be capable of achieving that task *together*.

In high performing teams, members (6, 7):

- Understand their own and other members' roles and responsibilities
- Encourage contributions of all members and ensure that the views of new and junior members are taken into account
- Show respect for the role, expertise, competence and contributions of allied disciplines and healthcare providers.
- Respect the leadership of the team
- Have the shared goal of high-quality care for the patient
- Show a commitment to teamwork in the best interest of the patient
- Recognize they are important to the outcome of the task
- Feel confident to raise their voice or intervene.

It is wonderful when one finds oneself working in an effective team. Sadly, it is much more common to find oneself in a ***group***. Giddings and Williamson (9) created a fascinating table which very clearly reveals the differences between a team and a group, which we reproduce here;


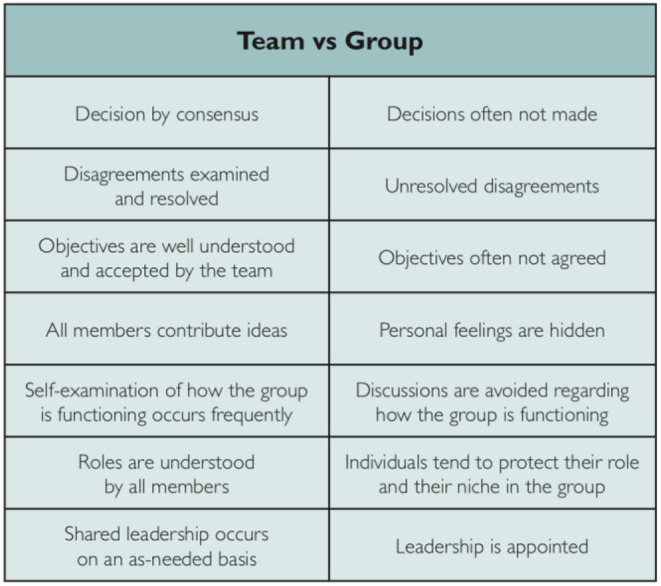


Some key points emerge from this table, and these are reinforced by our own experience and from watching other teams/groups in action. Consensus, clarity of goals and understanding of roles may seem obvious, but the hiding of feelings is perhaps less so. How many of us can remember being in meetings in which half the people in the room do not contribute to the decision making, but can be found moaning in the corridor about the decision that was made? This reflects a dangerous lack of confidence in not speaking up (always dangerous for patients) and a lack of leadership in bringing everyone's views to the fore. It does not do, either, to have a leader foisted on a team; better to let the team choose its leader and be ready to change leadership as and when circumstances change (and they always will). Good teams are self-analytical; happy to consider their own effectiveness and to make changes rapidly when required.

We are all aware that teams can be dysfunctional and have probably experienced situations in which an individual has thrown the train of successful teamwork off the tracks, derailing the team. Another great table from Giddings and Williamson (9) [based on the work of Hogan and Hogan (10)] looks at the characteristics of strong team members and those who tend to derail (see below). A quick glance down the “derailer” column usually prompts memories of specific individuals by anyone who reads it. It is also highly reminiscent of some world leaders at the time of writing! 
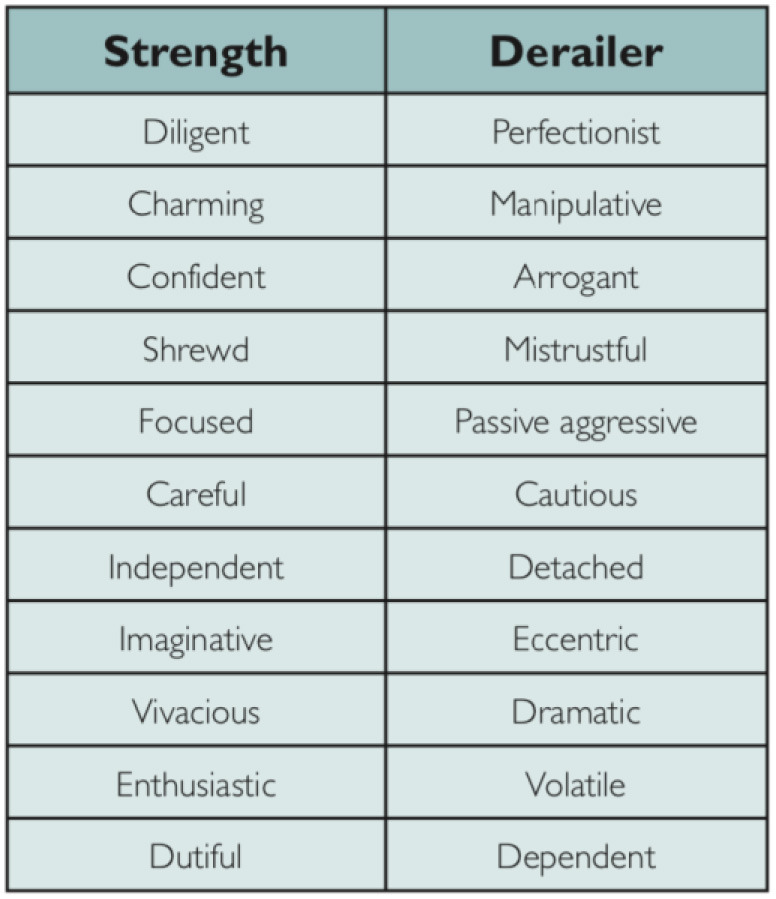


Teams in healthcare are often larger than is ideal, and leadership is critical for effective performance. There is evidence that leadership clarity in healthcare environments improves both teamwork and innovation (11). But what good leadership actually really means is harder to define, and it is salutary to think about it from the team members' perspective as Goodwin discussed (12). Team members define the most common positive attributes of *healthcare* leaders to be; intelligence, ability, confidence, warmth and friendliness, benevolence, emotional stability, integrity and the abilities to delegate and communicate. There seems nothing to argue about in this list. In our view, these are attributes to which leaders of teams in our field should aspire, and by which they should be judged.

## Issues Specific to Airway Teams

We have alluded to the wide membership of our own team in London, and it is worth considering why that should be necessary in a little more detail. As our team has evolved, the differences in core skills between the various members have become evident. This is best demonstrated by considering some examples.

- Within their discipline, ENT surgeons have developed skills in endoscopy (rigid and fibreoptic) and trans-endoscopic surgery. They are confident with very difficult airways and have had to work closely with specialist anesthetists to ensure the safety of their patients. They have specific technology and imaging experience and themselves often work in wider teams, for example in tracheostomy management. Culturally, ENT surgeons often receive direct referrals and stay in close contact with individual patients throughout the course of care, leading the management decisions.
- Cardiothoracic surgeons used to be just that, although it is becoming more common for cardiac and thoracic skills to be separated after appointment to the consultant (attending) staff, especially in pediatrics. Cardiac surgeons clearly are used to repairing the heart and blood vessels and to the use of cardiopulmonary bypass and ECMO. Both these latter can be lifesaving in severe airway problems. Cardiac surgeons are also used to working under time pressure because of the limitations of cardioplegic myocardial preservation, and to do so in a complex multi-disciplinary team of their own. Referrals usually are made to a team, via pediatric cardiologists and culturally almost all decisions are made in formal MDT meetings. Follow up of individual patients is often not directly with the surgeon, but by other members of the team, especially cardiologists and specialist nurses.
- Radiologists have a wonderful grasp of available technology, excellent dimensional interpretive skills and cross disciplines in their knowledge of diagnoses. In our world, this has led to developments in MR (flow dynamics), CT (4 D assessment of the airway), optical coherence tomography and advanced bronchography. The development of interventional radiology has involved them increasingly in therapy and follow up including endoscopic or physiologic imaging. The development of balloon dilatation of airway and image-guided surgery has further expanded the role of radiology in airway disorders. Culturally, radiologists have rarely had longer term follow up as part of their job description, with referral on each occasion usually being on a “form—request” basis. They have always been deeply involved in MDTs from a diagnostic perspective, but increasingly they are crucial members of the team in determining therapeutic options and the role in decision making is continuous.
- Pediatric cardiologists are integral to service delivery. Eighty percent of our patients have had cardiovascular problems, often complex, and cardiac diagnosis and non-surgical intervention fall within the realm of the cardiologists. They have a huge role to play in deciding the *timing* of interventions, and involvement in the MDT is essential for complex patients.
- All the above groups have a tendency to be “activist,” anxious to do something practical to intervene for the better. Such views need balancing, and the voice of the pediatric respiratory physician is crucial in this regard. It is often more valuable to the patient and his/her family to avoid surgery and the wisdom of someone who sees patients over time with detailed physiological and holistic assessment is of great importance to the team.
- The role of the nurse in the team cannot be underestimated. We have found it best to have a specialist “tracheal” nurse as the leader of the nursing team, and they have the front-line responsibility of communicating regularly with the families. It often comes as a surprise to surgeons that patients find them intimidating. This is rarely the case with nurses, who better grasp the wider needs of the patient and who take on a huge burden of communication with community services. Some of the most common complaints made against hospitals relate to difficulty in contacting the relevant person or failing to be called back with detail when promised. This is exactly what a well-trained and sympathetic nurse does well. Communication is everything.
- Administrative staff are needed to oil the wheels of the machine. The quality of service is dependent on them, and it is best to have them involved in all MDTs and team meetings. They ensure proper communication with other services and the family, and also help with maintaining the records and database, facilitating later research. They provide a great deal of support to families in practical, non-medical interactions with various authorities; many of these children have additional problems and appointments with hospital, school, social worker etc., all need to be coordinated to make a “one-stop shop” possible as often as possible. This is customer service. Some provide good service naturally, but it can be trained and should be expected.
- The remainder of the team comprises physio- and speech therapists, researchers and junior staff. Their role is variable, but they have much to offer; each will learn more about the patient and their voices should be heard.

Ross Brawn, the great Formula One manager, in describing what it takes to win a Formula One championship said (13)^2^ that “everyone in the team should aspire to be World Champion at what they do.” This is an important concept, reflecting disseminated ambition, strong leadership and a philosophy of excellence. Mostly, though it expresses the value of *every* member of a team in contributing to its success. People who fail to contribute to the team might better be employed elsewhere.

These are the human factors of surgery (14, 15), a field of study drawn from organizational psychology and of proven performance value in many industries, particularly aviation (16, 17). The team leaders should ensure that these human factors are monitored and appropriately maintained as time goes by. They need also to consider the impact of their own style (which can be measured) on others (18).

Most teams in medicine never have their team performance assessed. There are good examples of such assessment in certain specific areas, notably in anesthesia (19), emergency medicine (20), intensive care (21), and the operating room (22) in all of which there are good opportunities for simulation. The lack of assessment of complex teams with responsibilities for human lives is a situation that would not be allowed to exist in other high reliability organizations where regular human factors audit is regularly undertaken and is often part of licensing, for example the Line Operations Safety Audit to which commercial pilots and their teams are subject (16). Although not mandatory, it might be considered good governance for airway teams to subject themselves to such review.

## The Evolution of Airway Teams

Medicine is changing fast. The impact of technology, particularly in imaging, minimally invasive surgery and communications has been immense and is accelerating. Changing patterns of referral change the demands on the team as time passes. Teams should not be static in such a context. Membership should be reviewed; the unnecessary should be redeployed and new relationships fostered as demands change.

In our own team two developments over the last decade have driven the need for change. The first has been the development of tracheal transplantation in various forms (23, 24) and the increased incidence of button battery injury (25). For the first, we needed to involve a wide range of scientists, adult research teams and international contacts. They worked at multiple institutions but were able to join our MDTs and research meetings thanks to video conferencing. Not only was this necessary to manage the individual patients, but it added skills we lacked, and which have subsequently become integral. Notably, the integration of clinicians caring for adults helped us better to plan the *transition* of care from pediatric to adult practice and ensure the lifelong follow up necessary to determine the value of any intervention (26). For the second, close collaboration was needed with general pediatric surgeons with primary responsibility (and understanding) of the esophagus and its surgery.

Our team has thus changed its structure and now its name as a result. It is called the **aero-digestive team**. This reflects the changing pattern of work, and also how joint enterprise in a team format has meant that previously untried techniques are being developed to solve major problems (25), based on the respective skills of the team members. If close integration into team activities had not occurred, and if treatment was based on referral rather than working together as one team, these advances are unlikely to have been made. Once again, referrals are increasing, and the benefits of specialization are evident. The more you do, the better you get.

## The Future

There is an old Chinese proverb^3^ which goes “Those who have knowledge don't predict. Those who predict, don't have knowledge.” Despite the dangers of prediction, some things are coming our way and we need to anticipate them. There is no doubt that the advent of artificial intelligence and machine learning will have an impact on decision making and outcome analysis. Data are critical to both, and there can be no excuse in the current era for incomplete or inaccurate data collection. Transparency is necessary, and no patient should be excluded from the databases, unless they specifically refuse consent. Asked correctly, and with the altruism of involvement in future benefit, refusal is rare.

Augmented reality is developing so fast that many of the interventional procedures we employ will be supported by these techniques, as too will surgical learning and patient understanding. Combining these developments will permit simulation and improved precision. The development of all though will need “volume.” Research is unlikely to be funded for small or unstable teams.

Thus, there seems to be a significant rationale for centralizing pediatric airway care around teams of a certain, but undetermined, size and which embrace all the relevant skills. Such teams will benefit from international collaboration with similar teams, especially those of appropriate size. These teams exist and are contributing (27, 28). Research, transparency, partnership and co-operation are critical. As we said at the start of this paper, this is not a sport for individuals but for teams. Yet Brawn remains correct (13); if you want to win as a team, all the individuals must perform to the highest standard.

## Author Contributions

ME wrote the first draft, and all the other authors contributed to the final text and approved it for publication.

## Conflict of Interest

The authors declare that the research was conducted in the absence of any commercial or financial relationships that could be construed as a potential conflict of interest.

## Footnotes

^1^GOSH = The Great Ormond Street Hospital for Children NHS Foundation Trust, London.

^2^Paraphrased.

^3^Lao Tzu, 6th Century BC Chinese Poet.
